# Supplementary material for: Overexpression of Medicago sativa LEA4-4 can improve the salt, drought, and oxidation resistance of transgenic Arabidopsis
Source: PLoS One. 2020 Jun 4;15(6):e0234085. doi: 10.1371/journal.pone.0234085 (PMC7272090; doi:10.1371/journal.pone.0234085)
Supplement: S1 Table — (DOC) [file pone.0234085.s001.doc]

**S1 Table** Primers used in the study

| Primer | Primer sequence (5′→3′) | Application |
| --- | --- | --- |
| 3’ RACE | AAAGAATGGCATCCCACGACC | *MsLEA4-4* cloning |
| 5’ RACE | CTCAATCAACAACGTTACGACGG | *MsLEA4-4* cloning |
| *MsLEA*4-4-GFP-F | TACTCGAGATGGCATCCCACGACCAAAGT | Vector construction |
| *MsLEA*4-4-GFP-R | GCACTAGTATCAACAACGTTACGACGGGTTG | Vector construction |
| *MsLEA*4-4-3301-F | TGACCATGGTAGATCTATGGCATCCCACGACCAAAGTTA | Vector construction |
| *MsLEA*4-4-3301-R | ATTCGAGCTGGTCACCTCAATCAACAACGTTACGACGGGT | Vector construction |
| *npt*II-F | ATGATTGAACAAGATGGATTGCACGCAG | Genotyping |
| *npt*II-R | TCAGAAGAACTCGTCAAGAAGGCGA | Genotyping |
| 35S-3301-F | AACAGAACTCGCCGTAAAGACT | Genotyping |
| 35S-3301-R | CGGGACTCTAATCATAAAAACC | Genotyping |
| *MsLEA*4-4-F | TGATGGGCAACATAGGTGACAA | qRT-PCR |
| *MsLEA*4-4-R | ATTGGGCAGTTTCTTTGGTAGC | qRT-PCR |
| *AtActin*-F | GGACAAGTTATCACCATCGG | qRT-PCR |
| *AtActin*-R | TCAGCAATACCTGGAAACATAG | qRT-PCR |
| *MsActin*-F | GAGCGTTTCCGTTGTCCTGA | qRT-PCR |
| *MsActin*-R | AGGTGCTGAGGGAAGCCAAA | qRT-PCR |
| *ABI*5-F | GGAGGGCTAAGGGGAAGGA | qRT-PCR |
| *ABI*5-R | TAGGTGTCTTTACCTGTTGCTT | qRT-PCR |
| *ABF*3-F | CAGAAGAAACAAGTTGAAATCATGG | qRT-PCR |
| *ABF*3-R | CGTCAATGTCCTTCGCAAGC | qRT-PCR |
| *NCED*5-F | GGACAACTAAGCTCCGCCAT | qRT-PCR |
| *NCED*5-R | GATTTCTCACCTTCCGGCGA | qRT-PCR |
| *NCED*9-F | TCCGACATTCAAACCACCGT | qRT-PCR |
| *NCED*9-R | AGTTCCCGGCTATTTGGACG | qRT-PCR |
| *MsLEA*4-4 gsp1 | GGGCTGCTTGCTGGGTTTTGTCCTT | Genome walking |
| *MsLEA*4-4 gsp2 | CCTATGTTGCCCATCATCTGGTTTG | Genome walking |
| *MsLEA*4-4 gsp3 | GCTTTGTAACTTTGGTCGTGGGA | Genome walking |

Note: The underlined nucleotides indicate the restriction sites.
