# Supplementary material for: Overexpression of Medicago sativa LEA4-4 can improve the salt, drought, and oxidation resistance of transgenic Arabidopsis
Source: PLoS One. 2020 Jun 4;15(6):e0234085. doi: 10.1371/journal.pone.0234085 (PMC7272090; doi:10.1371/journal.pone.0234085)

**S1 Fig Constructing expression vector of *MsLEA*4-4.** (A) Diagram of the *MsLEA*4-4 overexpression vector. (B) Diagram of the *MsLEA*4-4-GFP expression vector.


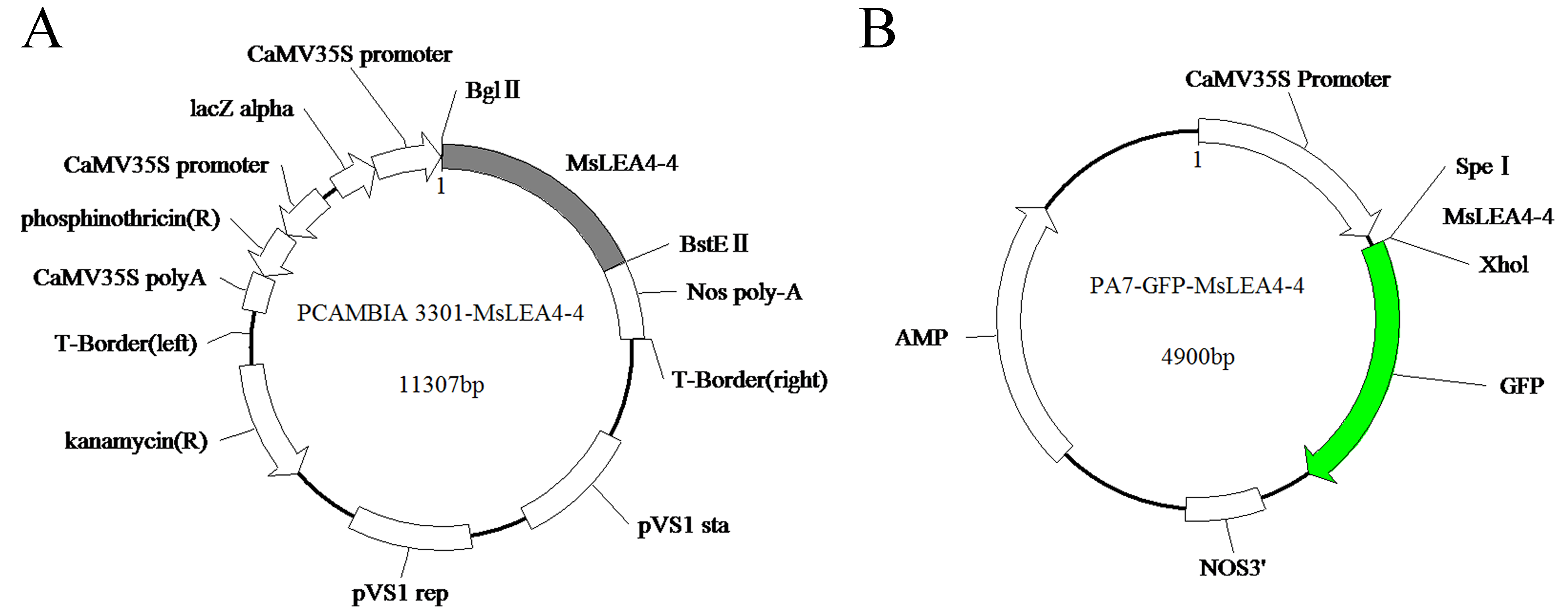

Supplement: S1 Fig — (A) Diagram of the MsLEA4-4 overexpression vector. (B) Diagram of the MsLEA4-4-GFP expression vector. (DOC) [file pone.0234085.s002.doc]
