# Supplementary figures and images for: Overexpression of Medicago sativa LEA4-4 can improve the salt, drought, and oxidation resistance of transgenic Arabidopsis
Source: PLoS One. 2020 Jun 4;15(6):e0234085. doi: 10.1371/journal.pone.0234085 (PMC7272090; doi:10.1371/journal.pone.0234085)

**S2 Fig Southern blot analysis of *MsLEA*4-4 overexpressed plants (OE1, OE4 and OE8).**

**
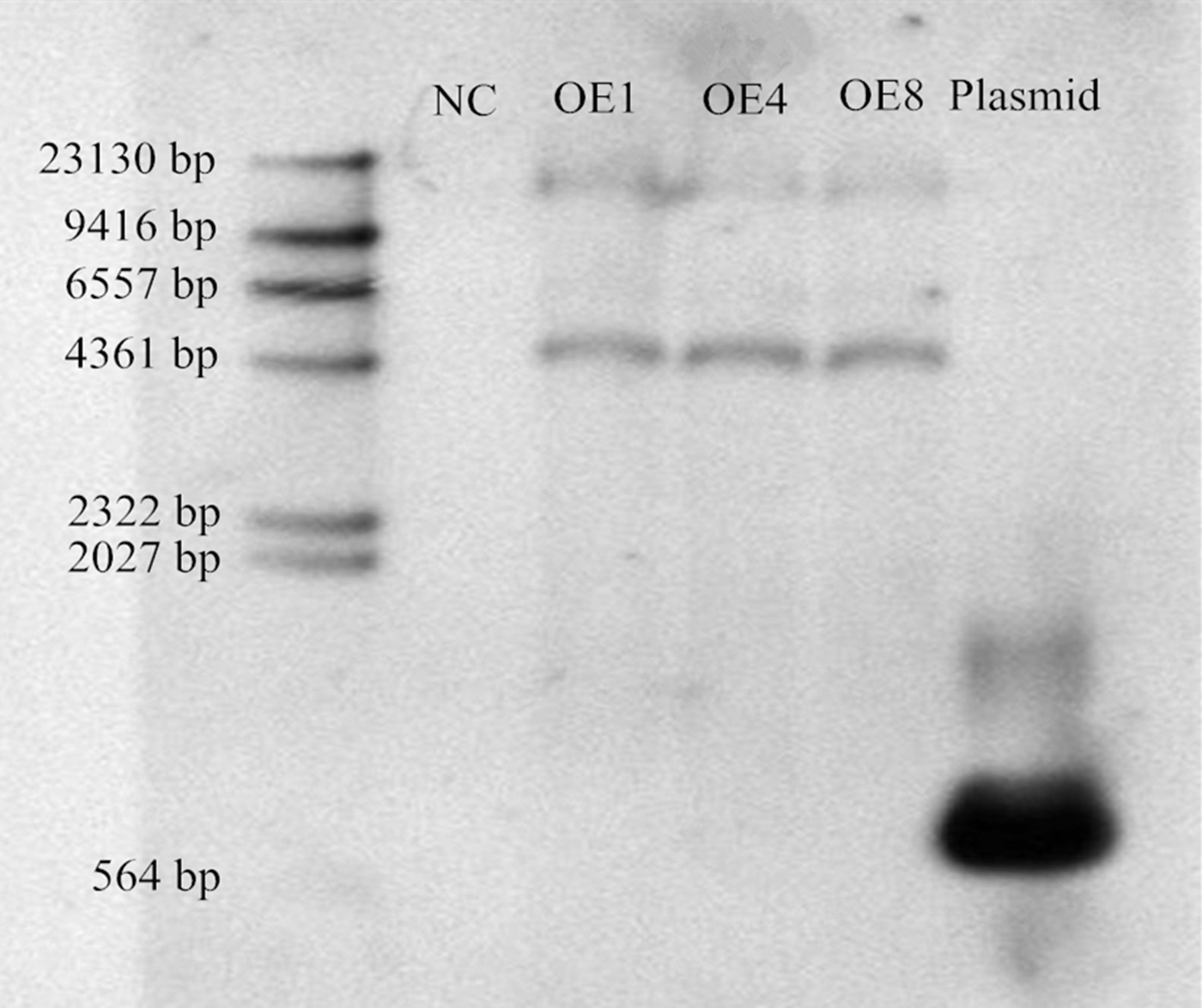
**

Supplement: S2 Fig — (DOC) [file pone.0234085.s003.doc]
