# Supplementary material for: Overexpression of Medicago sativa LEA4-4 can improve the salt, drought, and oxidation resistance of transgenic Arabidopsis
Source: PLoS One. 2020 Jun 4;15(6):e0234085. doi: 10.1371/journal.pone.0234085 (PMC7272090; doi:10.1371/journal.pone.0234085)

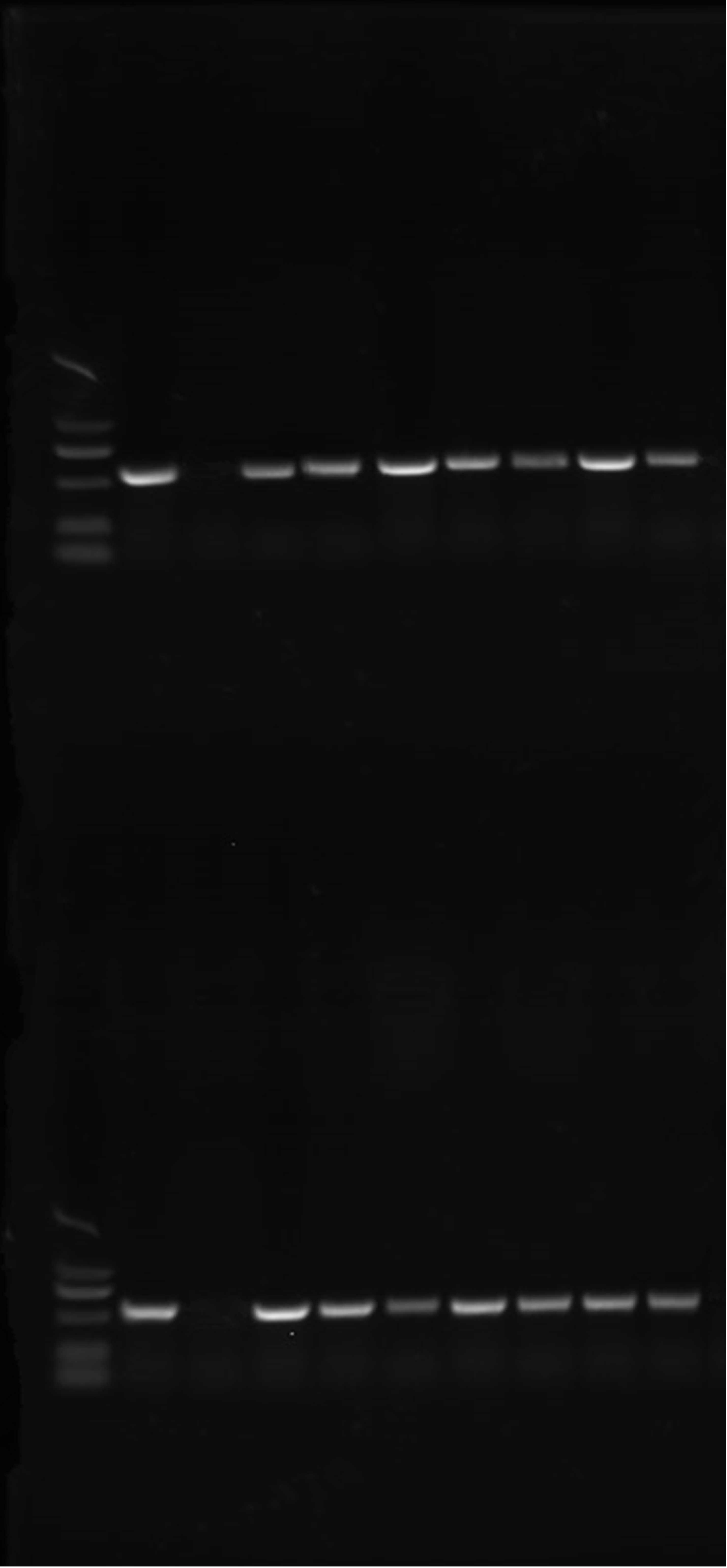

Supplement: S1 Raw Images — (PDF) [file pone.0234085.s004.pdf]
